# Spontaneous Local Calcium Transients Regulate Oligodendrocyte Development in Culture through Store-Operated Ca^2+^ Entry and Release

**DOI:** 10.1523/ENEURO.0347-19.2020

**Published:** 2020-08-14

**Authors:** Yanfang Rui, Stephanie L. Pollitt, Kenneth R. Myers, Yue Feng, James Q. Zheng

**Affiliations:** 1 Department of Cell Biology, Emory University School of Medicine, Atlanta, GA 30322; 2 Center for Neurodegenerative Diseases, Emory University School of Medicine, Atlanta, GA 30322; 3 Department of Pharmacology and Chemical Biology, Emory University School of Medicine, Atlanta, GA 30322; 4 Department of Neurology, Emory University School of Medicine, Atlanta, GA 30322

**Keywords:** Ca^2+^ signaling, internal Ca^2+^ stores, oligodendrocytes, store-operated Ca^2+^ entry

## Abstract

Oligodendrocytes (OLs) insulate axonal fibers for fast conduction of nerve impulses by wrapping axons of the CNS with compact myelin membranes. Differentiating OLs undergo drastic chances in cell morphology. Bipolar oligodendroglial precursor cells (OPCs) transform into highly ramified multipolar OLs, which then expand myelin membranes that enwrap axons. While significant progress has been made in understanding the molecular and genetic mechanisms underlying CNS myelination and its disruption in diseases, the cellular mechanisms that regulate OL differentiation are not fully understood. Here, we report that developing rat OLs in culture exhibit spontaneous Ca^2+^ local transients (sCaLTs) in their process arbors in the absence of neurons. Importantly, we find that the frequency of sCaLTs markedly increases as OLs undergo extensive process outgrowth and branching. We further show that sCaLTs are primarily generated through a combination of Ca^2+^ influx through store-operated Ca^2+^ entry (SOCE) and Ca^2+^ release from internal Ca^2+^ stores. Inhibition of sCaLTs impairs the elaboration and branching of OL processes, as well as substantially reduces the formation of large myelin sheets in culture. Together, our findings identify an important role for spontaneous local Ca^2+^ signaling in OL development.

## Significance Statement

While Ca^2+^ signals regulate a plethora of cellular activities and their spatiotemporal features, the role of Ca^2+^ signaling in oligodendroglia has not been well established. This study identifies a novel form of Ca^2+^ signaling, spontaneous Ca^2+^ local transients (sCaLTs) that play an important role in oligodendroglial development. In addition, this work reveals a new role for store-operated Ca^2+^ entry (SOCE) and release in generating sCaLTs and Ca^2+^ signaling in oligodendrocytes (OLs). Together, these findings establish a novel Ca^2+^ mechanism underlying oligodendroglial development.

## Introduction

Myelination of axons by oligodendrocytes (OLs) and Schwann cells in the central and peripheral nervous systems is essential for rapid conduction of nerve impulses (Nave and Trapp, 2008; Nave, 2010; Chang et al., 2016). Inflammatory demyelinating disorders, such as multiple sclerosis, affect over 400,000 individuals in the United States and are the most common disabling neurologic disease in young adults (Rolak, 2003). These neuroinflammatory diseases cause damage to the myelin sheath, thereby disrupting normal neuronal communication (Love, 2006; Alizadeh et al., 2015). In addition, aberrant myelin development also contributes to neuropsychiatric diseases represented by schizophrenia (Nave and Ehrenreich, 2014; Haroutunian et al., 2014; Mighdoll et al., 2015). Therefore, understanding the cellular mechanisms underlying the formation and maintenance of myelin is essential for developing therapies for treating demyelinating disorders.

During development, oligodendroglial precursor cells (OPCs) undergo drastic changes in their morphology. Multiple processes emerge from the cell body followed by extensive branching and outgrowth. These highly branched OL processes seek out target axons and enwrap them. From these branched OL processes, myelin membranes emerge and expand, finally compacting into lipid-rich myelin sheathes that insulate axons for fast nerve conduction. Each OL is capable of myelinating over 50 different axons (Baumann and Pham-Dinh, 2001; Snaidero and Simons, 2014), meaning that an OL must rapidly form and extend numerous branched processes, as well as generate large amounts of specialized membrane. Recent progress has been made toward understanding the process of axonal myelination (Bercury and Macklin, 2015; Nawaz et al., 2015; Hughes and Appel, 2016), and the identification of essential molecular components in OLs (Scherer and Arroyo, 2002; Miron et al., 2011). However, at the cellular level, there is still much to be learned about how OLs develop their complex morphology and myelinate axons.

Ca^2+^ is a key second messenger that regulates a diverse array of cellular activities, ranging from cell motility to gene transcription. The intracellular Ca^2+^ concentration ([Ca^2+^]_i_) of most cells at the resting level is maintained in the low nanomolar range. Spatiotemporally restricted fluctuations in [Ca^2+^]_i_ are the triggers for various cellular reactions and responsible for translating extracellular stimuli to specific cell responses. Ca^2+^ signaling is well studied in neurons for its essential role in synaptic transmission and the regulation of many, if not all aspects of neural development (Gomez and Zheng, 2006; Lohmann, 2009; Rosenberg and Spitzer, 2011; Sudhof, 2012). Ca^2+^ signaling and its potential functions in OLs, however, are not fully understood. Several studies have shown that voltage-gated Ca^2+^ channels (VGCCs) are expressed in OLs and demonstrated the elevation of [Ca^2+^]_i_ in response to ligand stimulation, neuronal activity, or injury (Kettenmann and Verkhratskii, 1994; Takeda et al., 1995; Haak et al., 2001; Soliven, 2001; Pitman and Young, 2016). More recently, it was shown that genetic knock-out of CaV1.2, an L-type VGCC, impairs OPC migration, process elaboration and myelination (Cheli et al., 2016; Santiago González et al., 2017). Ca^2+^ activity in OLs appears to play a critical role in OPC migration, OL differentiation, and myelin production *in vitro*, as well as controlling myelin sheath growth *in vivo* (Soliven, 2001; Cheli et al., 2015; Baraban et al., 2018; Krasnow et al., 2018). While these findings support a role for Ca^2+^ in OL development, the exact types of Ca^2+^ signals, their spatiotemporal patterns, mechanisms of generation, and their association with distinct stages of OL development remain unclear.

In this study, we report that developing OLs in culture exhibit spontaneous Ca^2+^ local transients (sCaLTs) that are largely restricted to discrete sites in branched OL processes. In particular, these sCaLTs are observed in purified rat OL cultures devoid of neurons. Importantly, the frequency of sCaLTs peaks in highly ramified OLs just before the formation of flat myelin sheets. Mechanistically, sCaLTs depend on store-operated Ca^2+^ entry (SOCE) and internal Ca^2+^ release. Finally, we present evidence that sCaLTs play an important role in OL development, especially in the elaboration of highly branched processes and the formation of myelin basic protein (MBP)-positive membranes in culture. Thus, these findings indicate an important role for spontaneous Ca^2+^ signals in OL development.

## Materials and Methods

### Primary culture of OL lineage cells

Rat mixed glial cell cultures were prepared following a previously published protocol (McCarthy and de Vellis, 1980). In brief, cerebral cortices from postnatal day 0–2 rat pups were dissected and mechanically dissociated. Dissociated cells were then cultured in poly‐L‐lysine‐coated 75‐cm^2^ tissue culture flasks with NM12 medium [high-glucose DMEM with 12% fetal bovine serum (FBS)]. The medium was fully replaced on days 3 and 7. At day 10, the flasks were shaken in a 37°C incubator for 45 minutes (min) at 50 rpm to remove loosely attached microglial cells. The flasks were then shaken overnight at 210 rpm in a 37°C incubator to dislodge OPCs from the astrocyte monolayer. The next morning, non-adherent cells were plated on an uncoated 10-cm tissue culture dish for 10 min to permit adherence of any residual microglia, while the loosely adherent OPCs were dislodged by gentle manual shaking. This step was repeated two more times to remove all microglia. The final supernatant from these shaken cultures contained ∼85–90% OPCs (Wilkins et al., 2001). The suspended cells were plated on poly-L-lysine coated coverslips at appropriate density (around 200,000 cells per 35-mm dish) in NM12 medium. After 6–8 hours (h), the NM12 medium was replaced by the FBS-free Super Sato medium composed of high-glucose DMEM with 2% B27-supplement, 1% horse serum, 110 μg/ml pyruvate, 50 μg/ml transferrin, 10 μg/ml insulin, 500 nm tri-iodo-thyronine, 520 nm L-thyroxine, and 2 mm GlutaMAX to induce differentiation. It was reported that neurons do not appear to survive this OL preparation protocol (McCarthy and de Vellis, 1980). Consistently, our immunostaining using Tuj1 antibody that recognizes neuronal β3 tubulin found no multipolar neurons, although a small number (<5%) of Tuj1-positive bipolar cells bearing short processes (one to two cell bodies in length) was observed. We therefore concluded that our OL cultures are essentially neuron free.

### DNA constructs and transfection

DNA constructs encoding pN1-Lck-GCaMP3 was a gift from Baljit Khakh (Addgene plasmid #26974; https://www.addgene.org/26974/; RRID:Addgene_26974; Shigetomi et al., 2010), and mRuby-Lifeact-7 was a gift from Michael Davidson (Addgene plasmid #54560; https://www.addgene.org/54560/; RRID:Addgene_54560). To express exogenous proteins in OLs, we used Lipofectamine 2000 (Invitrogen), following the manufacturer’s standard protocol, to transfect mixed glial cultures in 75-cm^2^ culture flasks 4 h before the overnight shaking step. We found that the transfection efficiency is much higher in OPCs grown in NM12 medium than in purified OLs grown in Super Sato medium.

### Antibodies and chemical reagents

The following antibodies and chemicals were used for this study: rabbit anti-α-tubulin (Abcam catalog #ab15246, RRID: AB_301787; 1:500), Rabbit anti-β3-tubulin (TUBB3; Covance catalog #MMS-435P, RRID: AB_2313773, 1:2000), mouse anti-MBP (BioLegend catalog #836504, RRID: AB_2616694; 1:1000), goat anti-Olig2 (R&D Systems catalog #AF2418, RRID: AB_2157554; 1:20), and mouse anti-O4 IgM (R&D Systems catalog #MAB 1326, RRID: AB_357617; 1:200). Alexa Fluor-conjugated secondary antibodies were all purchased from Thermo Fisher Scientific with a dilution of 1:500. nifedipine, verapamil, diltiazem, NNC 55-0396, ω-conotoxin GVIA (ω-CTX), ω-agatoxin IVA, SKF96365, cyclopiazonic acid (CPA), thapsigargin (Tg), and ryanodine (Ry) were all purchased from Sigma.

### CG4 cell line culture and differentiation

CG4 cells (Louis et al., 1992) were expanded in a proliferation medium consisting of DMEM with 1% heat inactivated FBS, insulin (5 μg/ml), transferrin (50 μg/ml), putrescine (100 mm), progesterone (20 nm), selenium (30 nm), biotin (10 ng/ml), PDGFAA (10 ng/ml), and bFGF (10 ng/ml). Differentiation of CG4 cells was induced by switching the cells from the proliferation medium to a differentiation medium containing DMEM, insulin (5 μg/ml), transferring (50 μg/ml), tri-iodothyronine (50 nm), and 0.5% FBS as previously described (Wang et al., 2004).

### RT-PCR

Total RNA from differentiated CG4 cells was extracted using Zymo Quick-RNA Miniprep Plus kit. Reverse transcription (RT) was performed using iScript cDNA Synthesis kit (Bio-Rad), followed by PCR and agarose gel analysis. The primers used for the PCR were reported previously (Latour et al., 2003; Li and Zhang, 2009; Avila et al., 2009) and are listed in Table 1.

**Table 1:** PCR primer sequences

Primer	Sequence	Temperature	Product size (bp)
Cacna1A-F	GAAAAGAGAGCCAGGGCTCTG	54	328
Cacna1A-R	CTGTTCTCGGGAGTCTTGGGG		
Cacna1B-F	GCTCGCTCTTCGTCTTCA	52	188
Cacna1B-R	AGGTTCCGTGTCATCCAGT		
Cacna1C-F	CCAGCCCAGAAAAGAAACAG	55	271
Cacna1C-R	ACTGCCTTTTCCTTAAGGTGCA		
Cacna1D-F	ATTGCCAGAAAAGAAAGCCTAGA	55	321
Cacna1D-R	GATGAGTTTGTGGCAACCCAC		
Cacna1E-F	ATGTCCCTGAAGATGTATGG	50	102
Cacna1E-R	AACGACCTCAAAGATGCTG		
Cacna1F-F	GACGGCAACTTGGCTTCT	53	144
Cacna1F-R	GCTGGCATGACTGCTGGT		
Cacna1G-F	CTGGAGAGGGCCAGGAGAGTCAGG	65	371
Cacna1G-R	GGCCGACCAGGAATCTCGCTCTC		
Cacna1S-F	ATGCCAGAGGATGACAACAAC	55	181
Cacna1S-R	CACCCAGAAAGACAATGATGAA		
Cacna1H-F	GGTTTGGGTACCATGAACTA	58	374
Cacna1H-R	GTAAACTCATAGACTCCGTG		
Cacna1l-F	TTATCTGCTCCCTGACTGG	58	406

### Immunostaining

Cells were grown on coverslips, fixed in 4% paraformaldehyde for 20 min, rinsed with PBS, permeabilized with 0.1% Triton X-100 for 5 min, blocked with 3% bovine serum albumin (BSA) for 1 h, incubated with primary antibodies diluted in 3% BSA overnight at 4°C, and then incubated with fluorophore-conjugated secondary antibodies at room temperature for 45 min.

### Microscopy and imaging

All imaging experiments were performed on an inverted Nikon Eclipse Ti-E microscope equipped with an automated z-drive with Perfect Focus and NIS-Elements software (Nikon). A 20× objective (Plan Fluor, 0.5 NA) was used for imaging fixed slices, and a 60× objective (Apo TIRF, 1.49 NA) was used for live-cell imaging. Two digital cameras were used for imaging acquisition: QuantEM 512SC CCD (Tyledyne Photometrics) and Orca-Flash four v.2 sCMOS (Hamamatsu). The QuantEM CCD offers the highest sensitivity for weak fluorescence but with only 512 × 512 resolution, whereas Flash4 sCMOS offers a balance between sensitivity and resolution. Both cameras offer a wide dynamic range with 16-bit digitization. For all the experiments, imaging settings were optimized such that the fluorescence signals are in the lower half of the camera dynamic range without any saturation. Presentation of the 16-bit digital images is done by using specific look up tables (LUTs) to illustrate the structures of interest.

#### Calcium imaging

OL lineage cells were loaded with 4 μm fluo-4-AM (Sigma) in Krebs–Ringer’s saline (150 mm NaCl, 5 mm KCl, 2 mm CaCl_2_, 1 mm MgCl_2_, 10 mm glucose, and 10 mm HEPES; pH 7.4; Bacci et al., 1999) at 37°C for 30 min, followed by three washes and a 15-min incubation period for further de-esterification of fluo-4-AM before imaging. Coverslips were mounted in a custom 35-mm live-cell chamber and maintained at 37°C with a heated stage adaptor (Warner Instruments). A 60× Apo TIRF objective (NA 1.49) and QuantEM 512SC were used. Time-lapse fluorescence images were acquired every 2 s.

##### Differential interference contrast (DIC) imaging

OL lineage cells on coverslips were mounted in a custom 35-mm live-cell chamber and maintained at 37°C with a heated stage adaptor. A 60× Apo TIRF objective (NA 1.49) and Orca-Flash4 camera were used. Time-lapse DIC images were acquired every 5 s. To measure the size of each OL from the DIC images, we connected the farthest tips of all the protruding branches, and then measured the total area of the resulting polygon.

##### For Lck-GCaMP3 and Ruby-Lifeact imaging

Mixed glial cells at day 10 were co-transfected with Lck-GCaMP3 and Ruby-Lifeact using Lipofectamine 2000 on 75-cm^2^ culture flasks 4 h before shaking, but otherwise OLs were differentiated and cultured as described above. OLs on coverslips were mounted in a 35-mm chamber and maintained at 37°C with a heated stage adaptor. A 60× Apo TIRF objective (NA 1.49) and Flash4 camera were used. Time-lapse fluorescence images were acquired every 2 s for Lck-GCaMP3 and 10 s for Ruby-Lifeact.

### Quantitative analysis of spontaneous Ca^2+^ local transients

To quantify individual sCaLTs, each time-lapse fluorescence (F) sequence was processed to generate a ΔF/F_0_ time-lapse sequence using ImageJ. Here, ΔF was generated using the “δ F up” plugin in ImageJ and F_0_ was the first fluorescent image without sCaLTs in the sequence. A maximal intensity projection of this ΔF/F_0_ time-lapse sequence was used to identify the discrete sites of sCaLTs, as well as to define the regions of interest (ROIs) by thresholding above the signal of sCaLT free regions. These ROIs were then used for frame-by-frame analyses of the original F sequence to generate ΔF/F_0_ for each ROI. To eliminate the possibility of encountering sCaLTs in the first frame, we selected F_0_ from the frame with the lowest signal within the first 1 min imaging for each ROI followed by background subtraction. We scored sCaLTs as increases of ΔF/F_0_ equal to or >20% of the baseline. We defined the frequency of sCaLTs as the number of sCaLTs observed in a 2-min period and measured the amplitude as the ΔF/F_0_ changes over the baseline. Data from all different regions of one cell was pooled together to generate one data point, at least four dishes from different batches of cultures were pooled together and analyzed for statistically significant differences using one-way ANOVA with *post hoc* tests. Compiled data are expressed and graphed as mean ± SEM, with *n* denoting the number of cells studied.

To better depict the spatiotemporal dynamics of sCaLTs in individual OLs, we generated a temporal-color coded image from a ΔF time-lapse sequence using ImageJ (image -> hyperstacks -> temporal-color code). In this temporal-color coded image, sCaLTs occurring at different time points are visualized in distinct colors as indicated on the color-coded scale bar, thus providing a visual summary of all the sCaLTs from the sequence.

### Quantitative analysis of changes in filamentous actin (F-actin) structures

We expressed Ruby-Lifeact in OLs to examine and quantify the dynamic changes in F-actin structures. Lifeact is a 17-amino acid peptide that labels F-actin structures in eukaryotic cells without interfering with actin dynamics *in vitro* or *in vivo* (Riedl et al., 2008). The fluorescence intensity of Ruby-Lifeact at a specific subcellular location depends on the local amount of F-actin, thus a change in Ruby-Lifeact signals at the particular location represents a change in F-actin content (e.g., from polymerization). At the same time, the area highlighted by Ruby-Lifeact will allow the measurement of the changes in actin-based cell protrusion. We performed a 20-min time-lapse imaging on OLs expressing Ruby-Lifeact with a 10-s frame interval. The maximal intensity projection of the Ruby-Lifeact sequence was produced and then divided by the first frame to result in a ratiometric image. This final ratiometric image depicts the increases in Ruby-Lifeact fluorescence (indicating increased F-actin polymerization) as well as new actin-based protrusions over the 20-min period, which we considered as the total F-actin changes. The ratio for areas without any changes in Ruby-Lifeact (including the background) is 1.00. We therefore measured the areas with the fluorescence ratio above the baseline of 1 and then normalized against the total area of the cell from the first frame to derive the F-actin change index.

### Morphologic analysis of OLs

#### Fractal dimension analysis

As previously described (Lourenço et al., 2016a), we used the fractal dimension (D) analysis to evaluate morphologic complexity of OLs by rendering a numerical value close to 1 for cells with low morphologic complexity (essentially bipolar cells) and near two for those with high complexity (highly branched cells). D value was calculated using the ImageJ software v1.51s (NIH). In detail, the fluorescence microscopy image was converted to 8 bit and then using the Crop tool, one cell was cropped, and the Threshold was adjusted to select the whole cell. Finally, the cell was outlined using the Outline tool and in Analyze, the Tools command was chosen. Next, the Fractal Box Count was selected and the corresponding D value was obtained. This procedure was performed for at least 50 cells per condition from each of at least three independent experiments.

#### Area of OL process arbors

The area of all OL processes/branches were measured from immunofluorescent signals of α-tubulin. Here, a threshold was applied to select all α-tubulin signals, and the total area was measured and subtracted by the cell body area. The results from 50 cells per three independent experiments were averaged and subjected to statistical analysis.

### Statistical analysis

All data from this study were collected from at least three replicates of independently prepared samples. We first assessed the normality of our data using histogram combined with Shapiro–Wilk test. Data following a normal distribution with only two conditions were analyzed using a two-tailed unpaired Student’s *t* test. Data following a normal distribution with three or more conditions were analyzed using one-way ANOVA with Tukey’s *post hoc* test. For data not following a normal distribution, a Kruskal–Wallis one-way ANOVA with a Dunn’s multiple comparison test was used for analysis. IMB SPSS Statistics 26 and Microsoft Excel were used for statistical analysis. Detailed statistical results, including *p* values, are provided in the corresponding figure legends. Data are presented as the mean ± standard deviation (SD) or mean ± standard error of the mean (SEM) as stated in text and in figure legends.

## Results

### sCaLTs in primary cultured rat OLs

**Figure 1.**
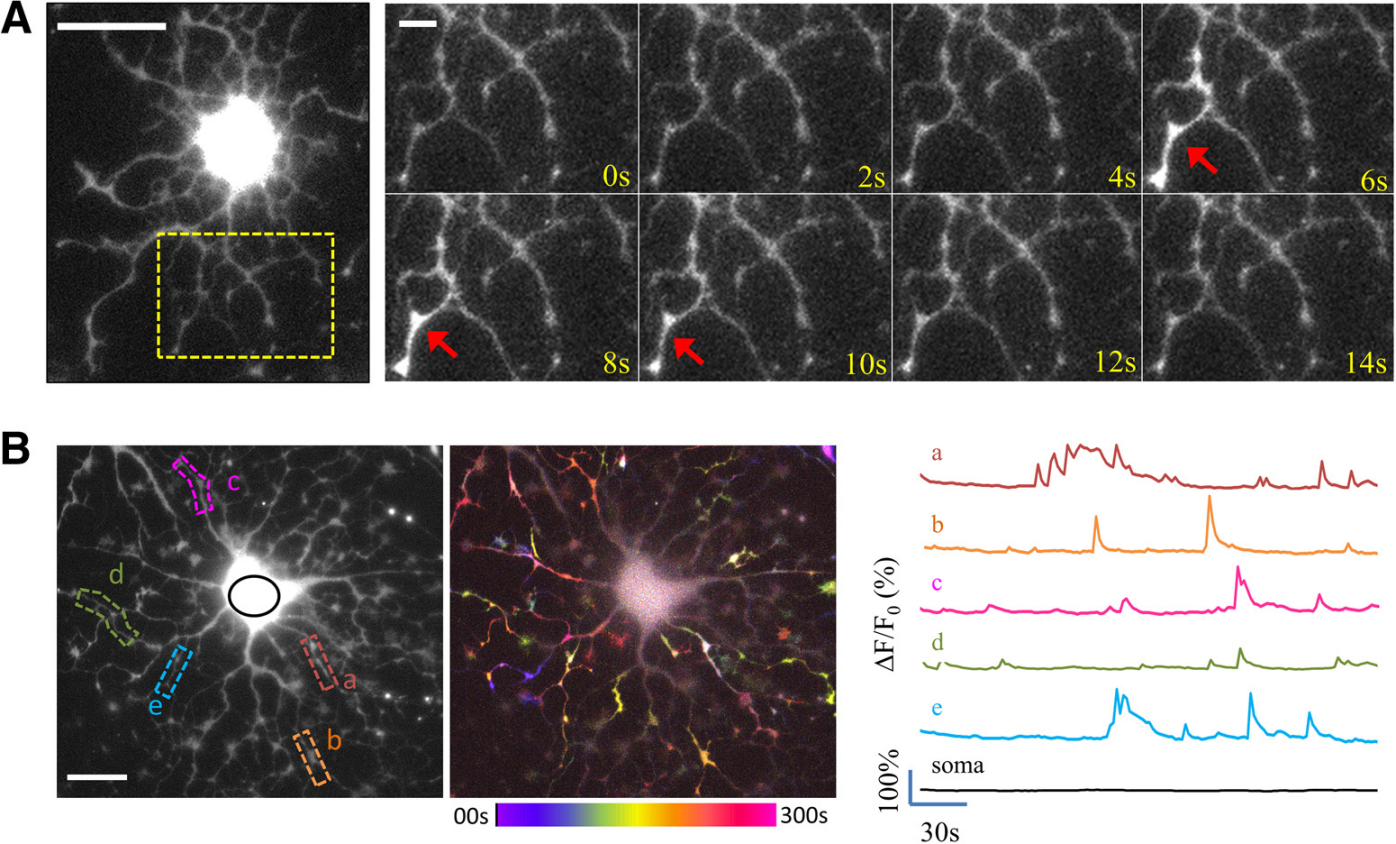
sCaLTs in primary cultured OLs. ***A***, A representative example of a sCaLT in a DIV2 rat OL labeled with fluo-4. The highly branched processes of this OL are clearly depicted in the fluorescent snapshot on the left. The time-lapse sequence of a small region containing multiple branches (dashed rectangle) is shown on the right. Red arrows indicate a sCaLT. Scale bars: 20 and 5 μm, respectively. ***B***, The widespread and transient nature of sCaLTs in cultured OLs. The left fluorescent image depicts a multipolar OL labeled with fluo-4 with five selected local regions displaying sCaLTs. The right fluorescent image is the same cell overlaid with the pseudocolored temporal-code map to depict changes in the fluo-4 fluorescence (ΔF) over the entire time-lapse period. The color bar depicts the colors associated with specific times in the sequence. Traces of ΔF/F_0_ of the five selected local segments, as well as the soma, are shown on the right. Scale bar: 20 μm.

Recent studies have indicated an important role for Ca^2+^ in axon myelination by OLs (Cheli et al., 2015; Baraban et al., 2018; Krasnow et al., 2018). Here, we performed live cell Ca^2+^ imaging to examine whether cultured OLs without neuronal contacts exhibit any fluctuations in their [Ca^2+^]_i_ at different stages of differentiation. We found that multipolar OLs, loaded with the Ca^2+^ indicator fluo-4, exhibit spontaneous Ca^2+^ transients that only occur in OL processes. Termed sCaLTs, these Ca^2+^ transients typically last for a few seconds and are spatially restricted to small segments of OL processes (Fig. 1*A*). The cell body was brightly labeled by fluo-4 due to its large volume but showed no change in fluorescence intensity. When examined over time, sCaLTs were observed in most of the OL processes, but not in the cell body (Fig. 1*B*). Here, the fluo-4 time-lapse sequence was processed to generate a pseudocolored temporally coded image (hereafter referred to as the temporal-code map) using the changes in fluo-4 intensity between frames (ΔF). The local and discrete nature of sCaLTs in multipolar OLs is clearly demonstrated by the temporal-code map. In addition, sample line traces of several sCaLT ROIs (referred to as the sCaLT sites) show that each site often exhibits multiple sCaLTs (Fig. 1*B*).

**Figure 2.**
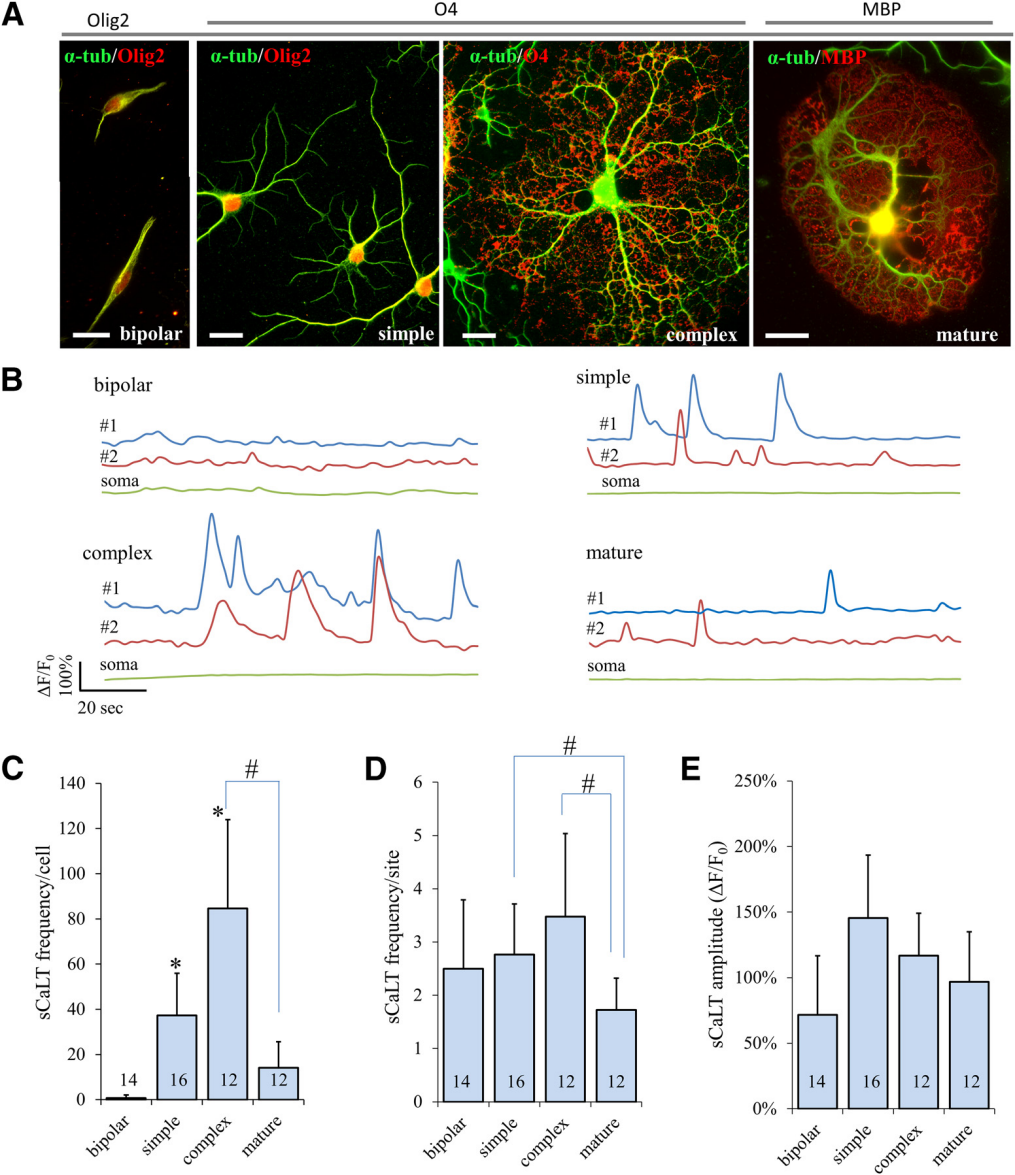
sCaLT frequency positively correlates with increased branch complexity during primary cultured OLs development. ***A***, Representative fluorescent images of four groups of OLs with distinct morphologies. Here, OLs were double stained for α-tubulin (α-tub; green) and OL markers (red: Olig2, O4, or MBP). Olig2 is present in all four groups of cells (OL lineage), O4 is expressed in both simple and complex cells (immature), and MBP is only expressed in mature cells. Scale bar: 20 μm. ***B***, Representative traces of ΔF/F_0_ showing sCaLTs in OLs from four different groups. Each colored line represents one ROI. ***C–E***, Bar graphs showing the sCaLT frequency/cell, sCaLT frequency/site, and sCaLT amplitude for each group of cultured OLs. Statistical analysis was performed using a Kruskal–Wallis one-way ANOVA with Dunn’s *post hoc* test in SPSS. Error bars represent the SD; *statistical difference (*p* < 0.05) from the bipolar group; #statistical difference (*p* < 0.05) between the two indicated groups. Numbers indicate the total number of cells examined for each group. It should be noted that only four of 14 bipolar cells displaying sCaLTs were quantified for the sCaLT frequency/site and amplitude. Quantification of sCaLT frequency/cell (Kruskal–Wallis test: *p* = 1.1882E-9, *H* = 44.49, df = 3; Dunn’s test: bipolar vs simple, *p* = 0.000012; bipolar vs complex, *p* = 2.1741E-9; complex vs mature, *p* = 0.001272). Quantification of sCaLT frequency/site (Kruskal–Wallis test: *p* = 0.002896, *H* = 14.007, df = 3; Dunn’s test: simple vs mature, *p* = 0.019867; complex vs mature, *p* = 0.002509). Quantification of sCaLT amplitude (Kruskal–Wallis test: *p* = 0.083240, *H* = 6.669, df = 3).

To determine whether and how sCaLTs change as OLs mature, we performed Ca^2+^ imaging on OLs at different stages of development according to the complexity of their morphology (Fig. 2*A*). We defined “bipolar” cells as those with only two processes spreading out from the cell body, “simple” cells as those having several processes without extensive tertiary branches, “complex” cells as those exhibiting highly ramified branches, and “mature” cells as those bearing large flat membranes (presumably the myelin sheets). To confirm the OL lineage and support our morphologic characterization of these cells, we stained them using the following OL markers: Olig2 (general OL lineage), O4 (for premyelinating OLs), and MBP (for mature OLs with myelin sheets). When fluo-4 time-lapse imaging was performed on these cells, we found that sCaLTs were only seen in simple, complex, and mature cells, but not in bipolar cells (Fig. 2*B*). To quantify sCaLTs at different stages of OL maturation, we counted the number of sCaLTs in a 2-min time period (Fig. 2*C–E*). Consistently, bipolar OPCs had essentially no sCaLTs, averaging a total of 0.71 ± 1.33 (mean ± SD, *n* = 14) sCaLTs per cell over the 2-min period (Fig. 2*C*). Of the fourteen bipolar cells we examined, only four had occasional sCaLTs. For the other three groups (simple, complex, and mature), every cell we examined displayed sCaLTs. The number of sCaLTs (referred to as the frequency/cell) was considerably higher in simple and complex cells than that in bipolar cells, averaging a total 37.31 ± 18.68 (mean ± SD, *n* = 16) and 84.67 ± 39.20 (mean ± SD, *n* = 12) sCaLTs per cell, respectively (Fig. 2*C*). The frequency/cell of sCaLTs in mature cells was 14.17 ± 11.51 (mean ± SD, *n* = 12). Since multiple sCaLTs were observed at each local site, we also calculated the number of sCaLTs per site over the 2-min window (referred to as the frequency/site). We found no difference in sCaLT frequency/site among the different groups except for the mature OLs, which showed a slight but significant reduction (Fig. 2*D*). No difference was detected in the average amplitude of sCaLTs (Fig. 2*E*). Together, these results show that sCaLTs frequency is positively correlated with OL elaboration of complex branches, thus suggesting a potential role for sCaLTs in OL development.

### The mechanisms underlying the generation of sCaLTs

**Figure 3.**
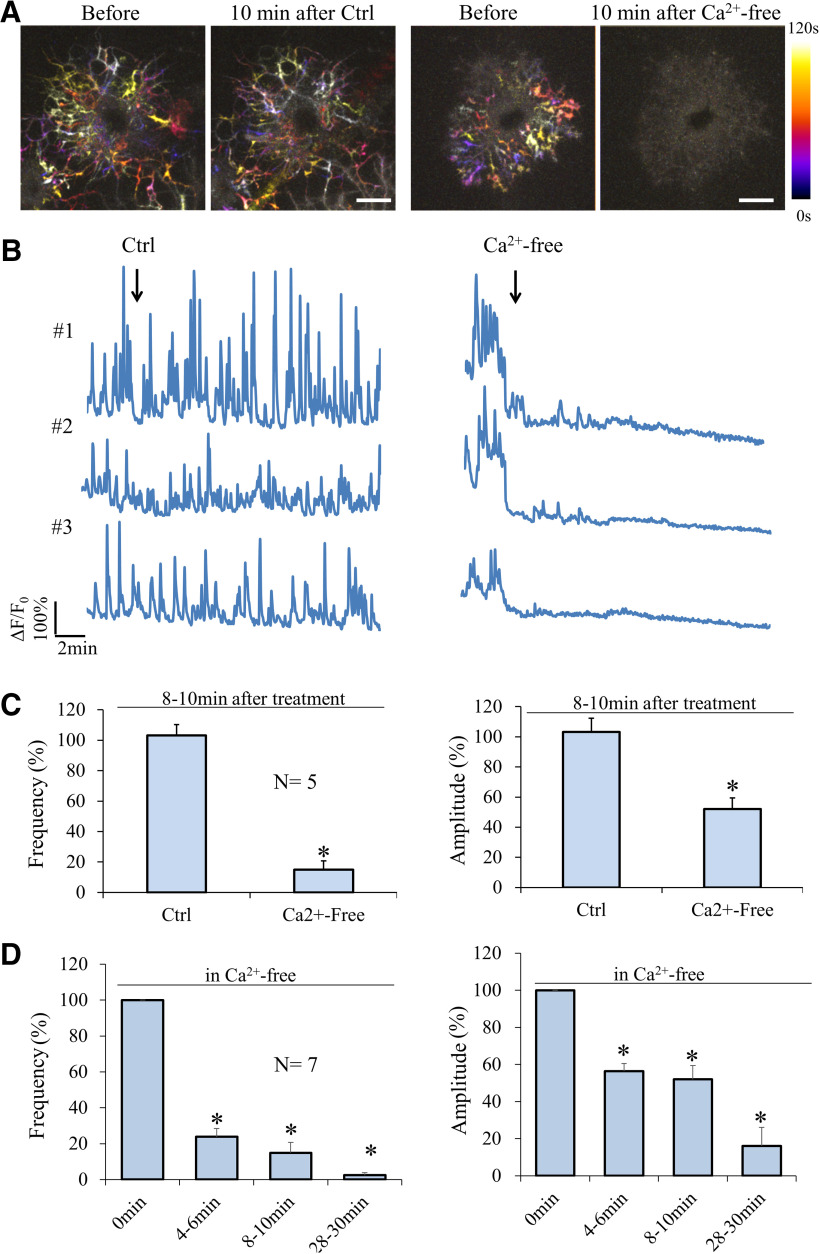
Removal of extracellular calcium abolishes sCaLTs. ***A***, Representative temporal-color maps showing sCaLTs before and after the addition of calcium-containing (Ctrl, left) or calcium-free (right) Krebs–Ringer’s solution. ***B***, Representative ΔF/F_0_ traces before and after application of calcium-containing (Ctrl, left) or calcium-free (right) Krebs–Ringer’s solution. ***C***, Bar graph shows the sCaLT frequency per cell (left) and amplitude (right) after 10-min treatment (normalized to the 2-min pretreatment values); *p < 0.05, Student’s *t* test. Error bars represent the SEM. *N*: the total number of cells examined. For frequency/cell, *p* = 1.45E-05; for amplitude, *p* = 0.002611. ***D***, Bar graph shows the frequency (left) and amplitude (right) at several time-points after application of calcium-free solution; *p < 0.05, one-way ANOVA with Tukey’s HSD test). Error bars represent the SEM. *N*: the total number of cells examined. Statistics on the frequency (df = 3, *F* = 134.295, *p* = 1.4868E-11; Tukey’s HSD test: 0 vs 4–6 min, *p* = 1.0008E-9; 0 vs 8–10 min, *p* = 1.9122E-10; 0 vs 28–30 min, *p* = 2.455E-11). Statistics on the amplitude (df = 3, *F* = 27.582, *p* = 0.000001; Tukey’s HSD test: 0 vs 4–6 min, *p* = 0.001232; 0 vs 8–10 min, *p* = 0.000476; 0 vs 28–30 min, *p* = 5.8439E-7).

Neurotransmitters released from neurons have been shown to elicit Ca^2+^ elevation in OLs (Takeda et al., 1995; Soliven, 2001; Butt, 2006; Nagy et al., 2017), but our primary OL cultures are devoid of neurons. Therefore, different mechanisms may underlie the generation of these spontaneous Ca^2+^ transients. We first examined the involvement of extracellular Ca^2+^ in sCaLTs and found that depleting extracellular Ca^2+^ using a Ca^2+^-free solution containing EGTA essentially eliminated sCaLTs (Fig. 3*A*,*B*). After a 10-min incubation in a Ca^2+^-free solution, the frequency of sCaLTs was reduced to 14.95 ± 5.85% (mean ± SEM) of the pretreatment period and the amplitude of remaining sCaLTs was reduced to 52.04 ± 7.41% (mean ± SEM) of the pretreatment period (Fig. 3*C*). We next examined the time course of changes in sCaLTs after depleting extracellular Ca^2+^ and found that marked reduction in both sCaLT frequency and amplitude was observed after 4-min incubation with the Ca^2+^-free solution (Fig. 3*D*). At 30 min, the frequency of sCaLTs was reduced to 2.43 ± 1.50% (mean ± SEM) of the pretreatment period (Fig. 3*D*). This result suggests that Ca^2+^ from the extracellular space is essential for the generation of sCaLTs in OLs.

**Figure 4.**
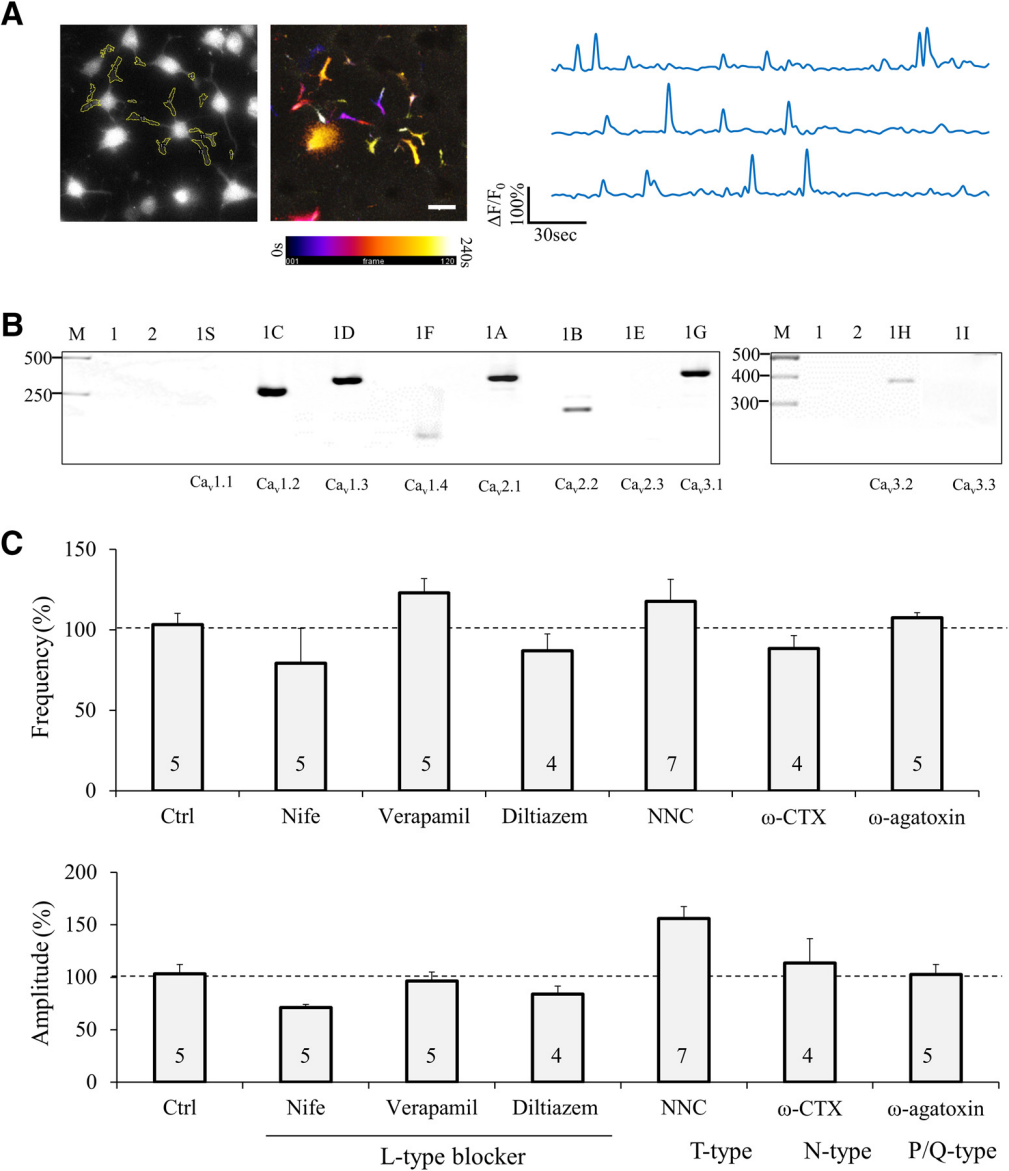
Expression of VGCCs, and the effects of VGCC inhibition on sCaLTs. ***A***, Differentiated CG4 cells exhibit sCaLTs similar to primary OLs. A representative fluo-4 image and the pseudocolor temporal-code map of sCaLTs are shown on the left. Sample ΔF/F_0_ traces of three selected regions are shown on the right. Scale bar: 20 μm. ***B***, RT-PCR showing the expression of several types of voltage-gated calcium channels in CG4 cells. Lane 1 is cDNA only control (no primer), lane 2 is water control (no cDNA), and rest lanes are samples with corresponding voltage-gated calcium channel primers. The voltage-gated calcium channel α1 subunit name and gene name were listed as follow: 1S (Ca_v_1.1; CACNA1S), 1C (Ca_v_1.2; CACNA1C), 1D (Ca_v_1.3; CACNA1D), 1F (Ca_v_1.4; CACNA1F), 1A (Ca_v_2.1; CACNA1A), 1B (Ca_v_2.2; CACNA1B), 1E (Ca_v_2.3; CACNA1E), 1G (Ca_v_3.1; CACNA1G), 1H (Ca_v_3.2; CACNA1H), and 1I (Ca_v_3.3; CACNA1I). Gel is representative of three independent experiments. ***C***, Bar graphs showing the effects of different VGCC blockers on the frequency/cell and amplitude of sCaLTs in primary OLs after 10-min treatment. Nife: nifedipine; NNC: NNC 55–0369. Error bars represent the SEM. Statistical analysis was done by one-way ANOVA with Tukey’s HSD test.

Since VGCCs have been implicated in OL development (Cheli et al., 2015), we examined whether VGCCs are responsible for the Ca^2+^ influx underlying sCaLTs. We first performed RT-PCR analysis to determine the types of VGCCs expressed in OLs. Because of the presence of other types of glial cells (e.g., astrocytes and microglia) in our primary OL culture, we elected to use a rat CNS glial precursor cell line (CG-4 cell) to obtain a large quantity of differentiating OLs (Louis et al., 1992) for RT-PCR analysis. It should be noted that differentiated CG4 cells exhibited sCaLTs, similar to primary OLs (Fig. 4*A*). Of the 10 VGCCs we examined, seven were detected in differentiated CG4 cells (Fig. 4*B*; see also Table 1 for the sequences of primers): L-type (α1C, α1D, α1F subunits), P/Q type (α1A), N-type (α1B), and T-type (α1G, α1H). Accordingly, we used specific pharmacological inhibitors to test the role of these VGCCs on sCaLTs in primary cultured OLs. These include L-type: diltiazem (100 μm), verapamil (100 μm), or nifedipine (10 μm); N-type: ω-CTX (1 μm); T-type: NNC 55-0396 (2 μm); and P/Q-type: ω-agatoxin IVA (100 nm). As summarized in Figure 4*C*, none of these specific blockers had significant effects on sCaLTs in OLs, suggesting that VGCCs are unlikely to play a major role in sCaLT generation. It should be noted that our results through acute blockage of VGCCs do not argue against a role for VGCCs in OL development as reported previously (Cheli et al., 2015, 2016), as genetic knock-out of specific VGCCs may impair OL development through complex and long-term mechanisms.

**Figure 5.**
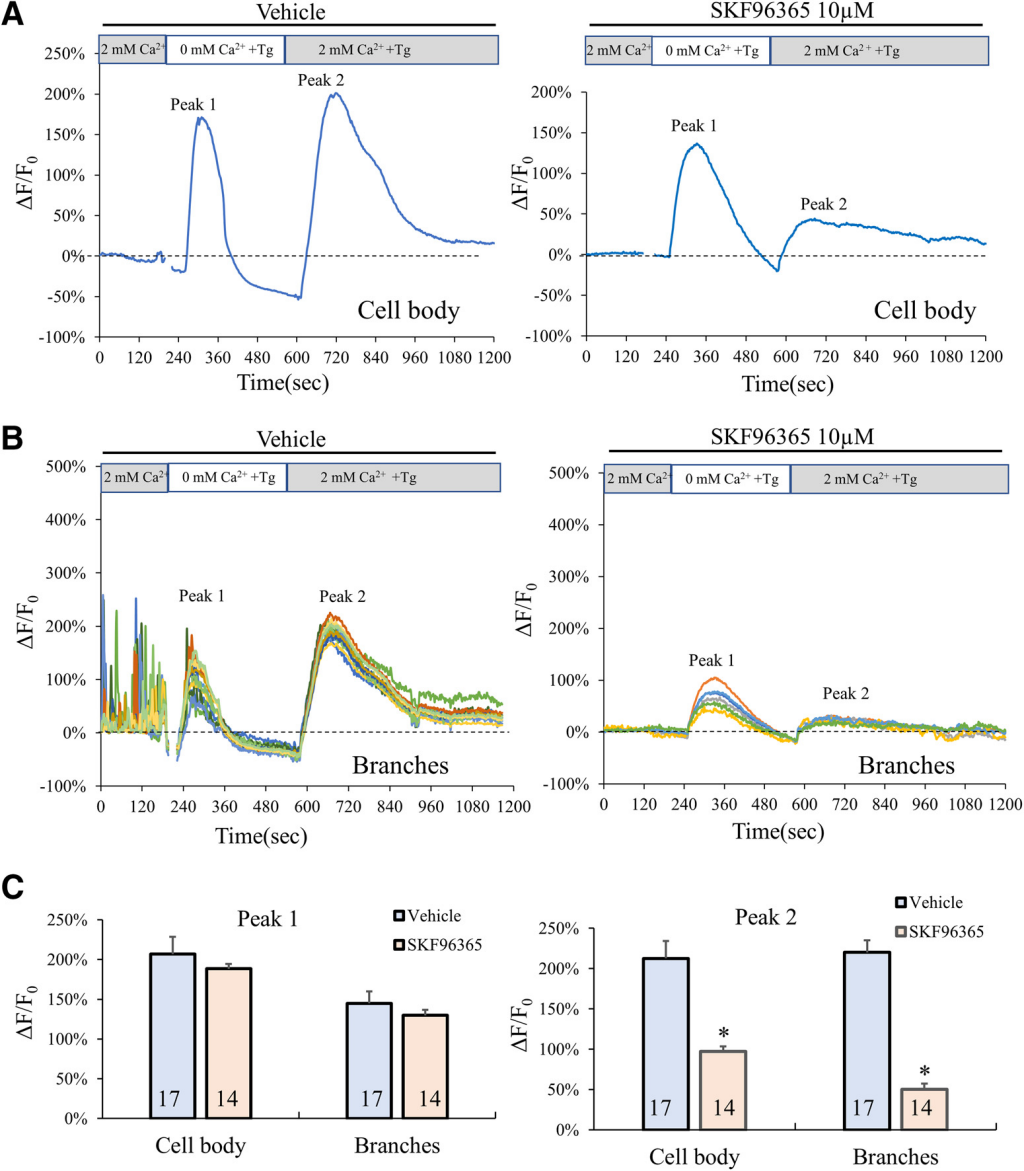
Presence of SOCE in OLs. ***A***, ***B***, Representative plot of the fluo-4 fluorescence (ΔF/F_0_) in the cell body (***A***) and in the branches (***B***) of an OL subjected to the following sequence of treatments: 2 min control in a 2 mm Ca^2+^ saline buffer, 8 min in a Ca^2+^-free buffer containing 1 μm Tg, and 10 min in a 2 mm Ca^2+^ saline buffer containing 1 μm Tg. ***C***, Bar graphs showing the average ΔF/F_0_ values (%) in cell body and branches for peak #1 and peak #2. *n* = number of cells from at least three independent experiments; *statistical difference (*p* < 0.05) when comparing SKF96365 application to vehicle control. All *p* values were calculated using two tail unpaired Student’s *t* tests. Peak #2: for cell body, *p* = 0.00528278; for branches, *p* = 3.52E-44.

SOCE is a part of the calcium homeostasis machinery and has been shown to play a role in Ca^2+^ signaling in a variety of cells (Prakriya and Lewis, 2015; Bagur and Hajnóczky, 2017). OLs express Orai and stromal interaction molecule (STM), two major components of SOCE (Hoffmann et al., 2010; Papanikolaou et al., 2017), but the function of these channels in OLs remain unknown. We thus examined whether SOCE is involved in the generation of sCaLTs in OLs. We used a standard protocol to test if SOCE is present in our cultured OLs (Papanikolaou et al., 2017; Hou et al., 2018). Here, fluo4-loaded OLs were first imaged in a control saline solution containing 2 mm Ca^2+^. Extracellular Ca^2+^ was then removed using a Ca^2+^-free buffer containing EGTA and Tg, a non-competitive inhibitor of the sarco/endoplasmic reticulum (ER) Ca^2+^ ATPase, to deplete the internal Ca^2+^ store. Consistent with previous studies, exposure of OLs to Tg elicited a large and transient elevation in cell body [Ca^2+^]_i_ due to the Ca^2+^ release from the internal store (hereafter referred as peak #1), which is followed by the resting [Ca^2+^]_i_ dropping to a lower level than that of the control due to the lack of Ca^2+^ in the extracellular space (Fig. 5*A*). Adding back 2 mm Ca^2+^ to the extracellular buffer elicited another large and transient elevation of [Ca^2+^]_i_ (hereafter referred to as peak #2), which, as previously described (Papanikolaou et al., 2017; Hou et al., 2018), is a result of SOCE activation. We confirmed this in our cells by attenuating Peak #2 using SKF96365, a STIM blocker (Fig. 5*A*; Merritt et al., 1990; Várnai et al., 2009). SOCE-mediated elevation in [Ca^2+^]_i_ was also observed in the OL branches where sCaLTs were observed (Fig. 5*B*), suggesting the presence of SOCE in OL branches. Quantitative analysis shows that SKF96365 (10 μm) was very effective in inhibiting the elevation in [Ca^2+^]_i_ (peak #2) in response to the depletion and reintroduction of extracellular Ca^2+^ (Fig. 5*C*). These data provide evidence that SOCE is present in OLs.

**Figure 6.**
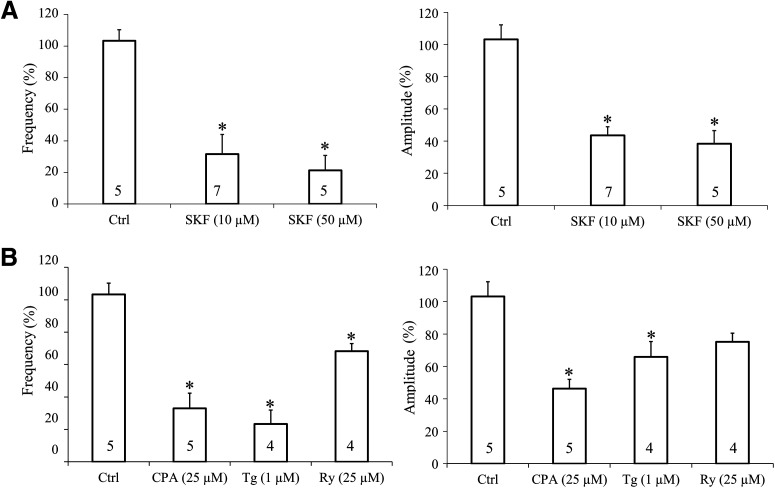
Inhibition of SOCE or internal calcium store attenuates the frequency and amplitude of sCaLTs. ***A***, Effects of SKF96365 (10 μm and 50 μm) treatment on sCaLT frequency (left) and amplitude (right) normalized to pretreatment baseline. *n* = number of cells from at least three independent experiments. ***B***, Effects of internal calcium store blockers on sCaLT frequency (left) and amplitude (right). SFK: SKF96365, CPA:cyclopiazonic acid, Tg: thapsigargin; Ry: ryanodine. *n* = number of cells from four independent experiments; *statistical difference (*p* < 0.05) for unpaired comparison to the baseline control period. All *p* values were calculated using one-way ANOVA with Tukey’s HSD test. For SKF96365, quantification of frequency (df = 2, *F* = 15.473, *p* = 0.000285; Tukey’s HSD test: Ctrl vs SKF 10 μm, *p* = 0.000871; Ctrl vs SKF 50 μm, *p* = 0.000521), quantification of amplitude (df = 2, *F* = 24.152, *p* = 0.000029; Tukey’s HSD test: Ctrl vs SKF 10 μm, *p* = 0.000122; Ctrl vs SKF 50 μm, *p* = 0.000050). For internal calcium store inhibitors, quantification of frequency (df = 3, *F* = 20.043, *p* = 0.000017; Tukey’s HSD test: Ctrl vs CPA, *p* = 0.000054; Ctrl vs Tg, *p* = 0.000040; Ctrl vs Ry, *p* = 0.046953), quantification of amplitude (df = 3, *F* = 11.182, *p* = 0.000413; Tukey’s HSD test: Ctrl vs CPA, *p* = 0.000209; Ctrl vs Tg, *p* = 0.018541; Ctrl vs Ry, *p* = 0.092622).

It should be noted that, in the presence of SFK96365, sCaLTs in the OL branches were largely diminished even when extracellular Ca^2+^ was present (Fig. 5*B*, control period). This suggests that SOCE may be the predominant pathway for sCaLT generation. We further analyzed the effect of SOCE inhibition by SKF96365 on sCaLTs in the presence of 2 mm extracellular Ca^2+^. We found that exposure to 10 μm SFK96365 for 10 min markedly reduced the frequency and amplitude of sCaLTs to 31.56 ± 12.50% and 43.56 ± 5.30% (mean ± SEM) of the control values, respectively (Fig. 6*A*). We next used cyclopiazonic acid (CPA, 25 μm), or Tg (1 μm), to inhibit the ER Ca^2+^ pumps and found that both inhibitors abolished the sCaLTs in OLs, even in the presence of extracellular Ca^2+^ (Fig. 6*B*). Furthermore, application of Ry (25 μm) also significantly reduced the frequency of sCaLTs. Together, these results indicate that Ca^2+^ release from the internal Ca^2+^ store coupled with SOCE underlie the development of sCaLTs in cultured OLs.

### sCaLTs in OL development and maturation

**Figure 7.**
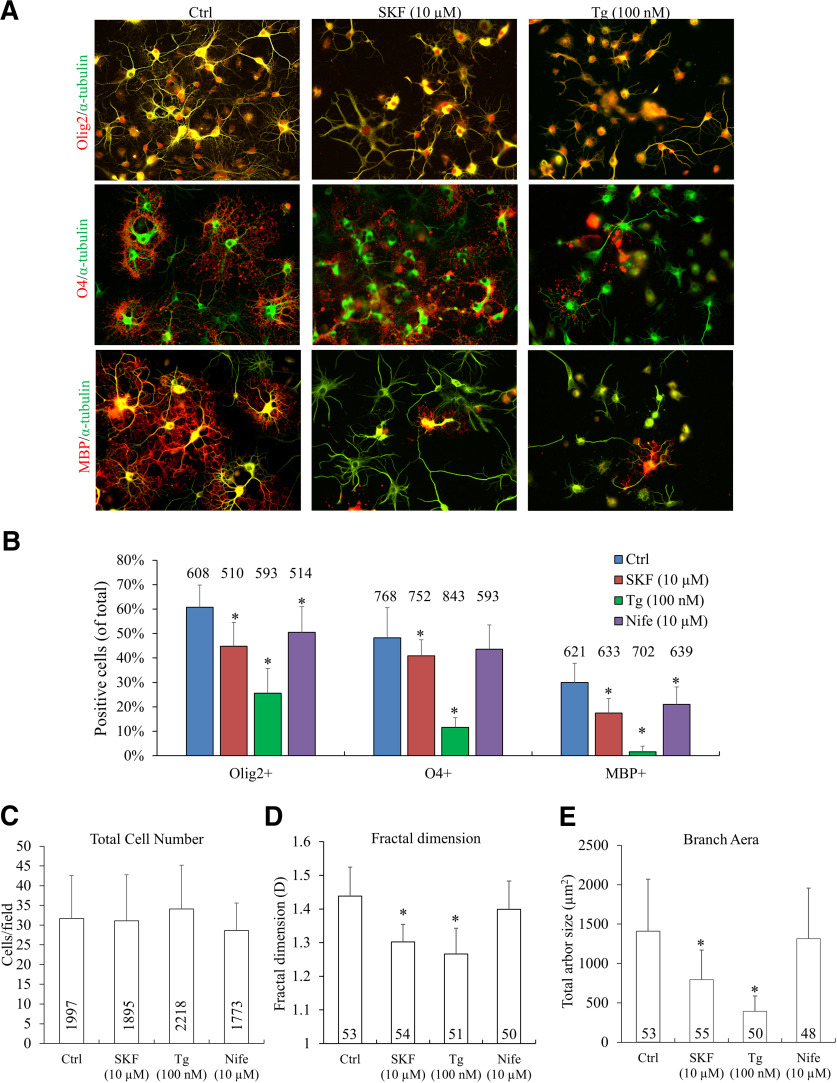
Effects of long-term (4 d) sCaLTs inhibition on OL differentiation and growth in culture. ***A***, Representative images of cells exposed to different calcium inhibitors. One day after plating, cells were treated with vehicle or inhibitors for 4 d. Cells were fixed at DIV5 and stained for α-tubulin (green) and OL markers (Olig2, O4, or MBP; red). Scale bar: 50 μm. ***B***, Bar graphs showing the percentage of cells positive for each OL marker after respective drug treatment. ***C***, Bar graphs showing the quantification of the total number of cells for each drug condition. ***D***, ***E***, Bar graphs showing D values (fractal dimension) and the total arbor size of OLs after each treatment. *n* = number of cells from at least three independent experiments; *statistical difference (*p* < 0.05) compared with the corresponding vehicle or control group (one-way ANOVA with Tukey’s HSD test). Error bars represent the standard error. Quantification of positive markers: Olig2+ (df = 3, *F* = 48.610, *p* = 9.841E-18; Tukey’s HSD test: Ctrl vs SKF, *p* = 0.000014; Ctrl vs Tg, *p* = 5.7843E-13; Ctrl vs Nife, *p* = 0.006978), O4+ (df = 3, *F* = 77.082, *p* = 1.5532E-25; Tukey’s HSD test: Ctrl vs SKF, *p* = 0.048013; Ctrl vs Tg, *p* = 4.8717E-13; Ctrl vs Nife, *p* = 0.396643), MBP+ (df = 3, *F* = 80.817, *p* = 2.0443E-24; Tukey’s HSD test: Ctrl vs SKF, *p* =1.8915E-8; Ctrl vs Tg, *p* = 5.3879E-13; Ctrl vs Nife, *p* = 0.000056), quantification of fractal dimension (df = 3, *F* = 59.285, *p* = 1.3288E-27; Tukey’s HSD test: Ctrl vs SKF, *p* = 4.5963E-13; Ctrl vs Tg, *p* = 4.5952E-13; Ctrl vs Nife, *p* = 0.064), quantification of surface area of OLs (df = 3, *F* = 45.033, *p* = 2.6408E-22; Tukey’s HSD test: Ctrl vs SKF, *p* = 1.0261E-8; Ctrl vs Tg, *p* = 3.9668E-13; Ctrl vs Nife, *p* = 0.786579).

We first examined the effects of inhibiting sCaLTs on OL differentiation in culture. Here, OLs after 1 day in culture [referred to as day *in vitro* (DIV)1] were treated with SKF96365, Tg, or nifedipine for 4 d and fixed on DIV5 for immunolabeling with specific OL markers Olig2, O4, and MBP (Fig. 7*A*). We found that treatment with SFK96365 (10 μm) and Tg (100 nm) markedly reduced the percentage of cells expressing these OL lineage markers (Fig. 7*A*,*B*). In particular, MBP-positive cells were significantly reduced from 29.9 ± 7.9% (mean ± SD; control) to 17.4 ± 6.0% (mean ± SD) and 1.6 ± 2.3% (mean ± SD) by SFK96365 and Tg, respectively (*p* = 1.2E-06 and *p* = 1.1E-13). Of the inhibitors used, Tg appears to have the most profound effect on OL maturation, indicating the importance of Ca^2+^ internal stores in OL development. Importantly, we did not observe any changes in the overall number of cells, suggesting that cell death was not induced by these pharmacological treatments (Fig. 7*C*).

We further analyzed the effect of these inhibitors on the morphologic complexity of OLs using fractal dimension (D) analysis as described previously (Behar, 2001; Fernández and Jelinek, 2001; Lourenço et al., 2016b). Hence, lower D values are typical of cells with low process branching while higher D values reflect a higher degree of morphologic complexity. OLs exposed to SKF96365 (10 μm) or Tg (100 nm) displayed a lower D than control cells, indicating a reduction in the morphologic complexity of these OLs (Fig. 7*D*). The reduction in morphologic complexity is further supported by a decrease in the total arbor size, as measured by the total area covered by the OL arbor minus the cell body (hereafter referred to as total arbor size (Fig. 7*E*). Exposure of OLs to 10 μm nifedipine appears to reduce the percentage of cells expressing Olig2 or MBP, but not O4 (Fig. 7*B*). However, 10 μm nifedipine has no effect on OL morphologic complexity and total arbor size (Fig. 7*C–E*), which makes it unclear if nifedipine affects OL differentiation. Overall, these findings suggest that SOCE and store Ca^2+^ release are the major players regulating the molecular differentiation and morphologic development of OLs in culture. It is plausible that sCaLTs generated through SOCE and store Ca^2+^ release, contribute to and regulate OL differentiation and maturation during development.

**Figure 8.**
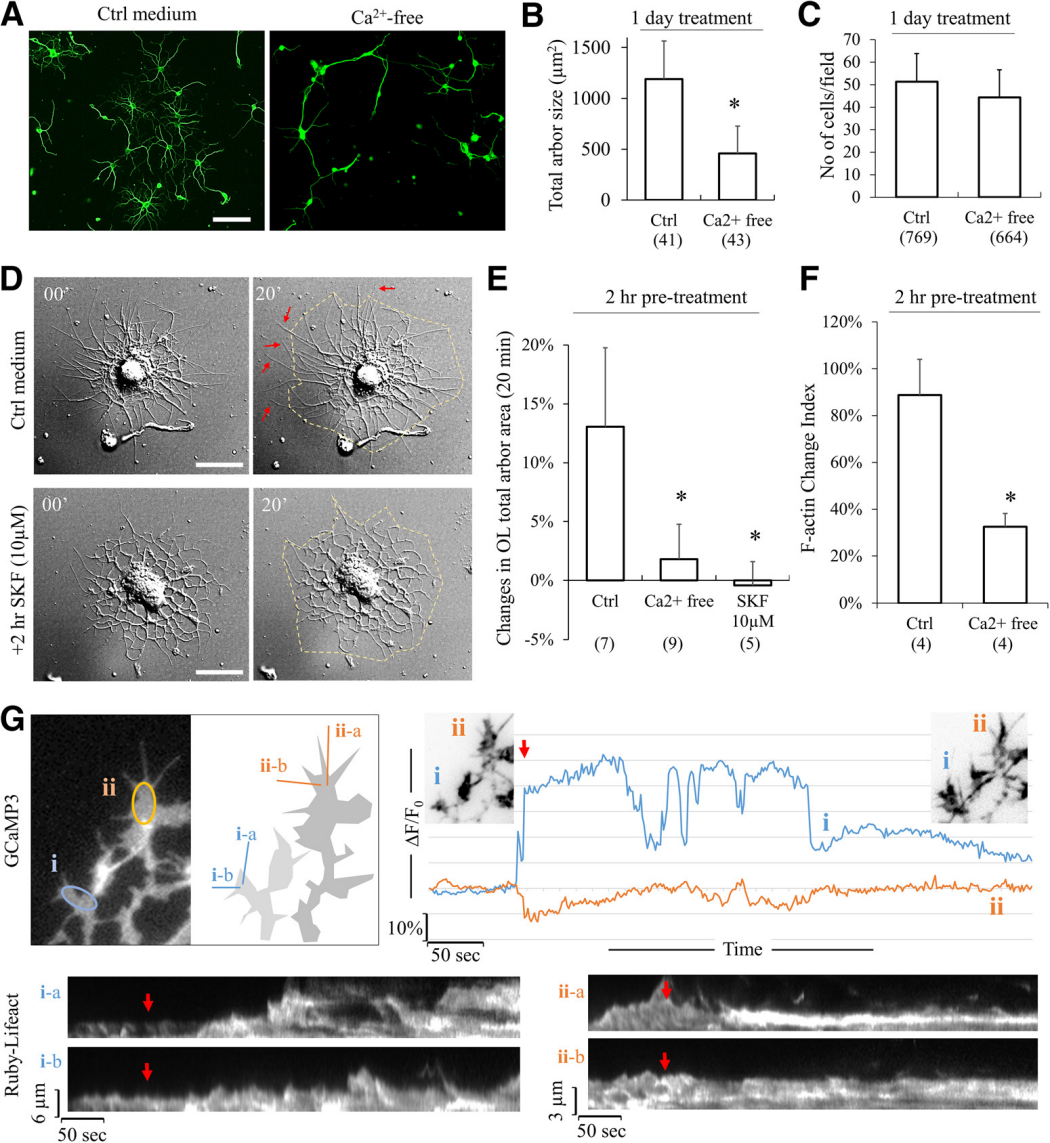
Effects of short-term (2 h and 1 d) sCaLTs inhibitions on OL differentiation and growth in culture. ***A***, Representative images of α-tubulin staining of cultured OLs incubated in control medium or Ca^2+^-free medium for 1 d (DIV3–DIV4). Scale bar: 50 μm. ***B***, The bar graph shows the total arbor size after being cultured in control medium or Ca^2+^-free medium for 1 d. Numbers in parentheses indicate the number of cells examined for each condition from three independent experiments. Error bars represent SD; *p < 0.05 (*p* = 1.045E-15). ***C***, The bar graph shows total cell number of OLs after being cultured in control medium and Ca^2+^-free medium for 1 d. Error bars represent the SD. Numbers in parentheses indicate the number of cells examined for each condition from three independent experiments. ***D***, DIC images showing the morphologic changes of OLs in 20 min after being pretreated with control medium or SKF 10 μm for 2 h. Scale bar: 20 μm. The quantification is shown as a bar graph in ***E***. Error bars represent the SD. Numbers in parentheses indicate the number of cells examined for each condition; *statistical difference (*p* < 0.05) compared with the corresponding control group (one-way ANOVA with Tukey’s HSD test). Quantification results (df = 2, *F* = 18.406, *p* = 0.000044; Tukey’s HSD test: Ctrl vs SKF, *p* = 0.000153; Ctrl *vs*. Ca^2+^-free, *p* = 0.000169). ***F***, Bar graph shows the F-actin change index for OLs incubated with control medium or Ca^2+^-free medium for 2 h. *n* = number of cells from four independent experiments. Error bars represent the SD; *statistical difference (*p* < 0.05, unpaired Student’s *t* test); *p* = 0.002759. ***G***, Association between sCaLTs and actin-based protrusive activity. The upper left pair of panels show a representative GCaMP3 fluorescent image of a part of an OL, which is shown as a schematic drawing on the right. Both GCaMP3 and Ruby-Lifeact were expressed in this OL. Two subregions of interest (i) and (ii) are indicated by the oval shapes and their changes in GCaMP3 fluorescence (ΔF/F_0_) are shown in the line plots on the right, which clearly shows that (i) region exhibited a sCaLT. The two insets in the line plots show the Ruby-Lifeact fluorescent images (inverted greyscale) of the same region before and after the sCaLTs. To examine the actin-based protrusive activities of these two subregions, two straight lines for each of the two subregions were used to generate the kymograph of Ruby-Lifeact signals shown at the bottom. Red arrows indicate the onset of the sCaLT in (i).

It should be noted that extended period of pharmacological treatment could generate unintended effects on OL development. Therefore, we performed a series of experiments in which DIV3 OLs were treated to block sCaLTs for only 24 h. We found that removal of extracellular Ca^2+^ using the calcium-free solution for 24 h resulted in a substantial reduction in OL branches (Fig. 8*A*,*B*), whereas no loss of cells was observed (Fig. 8*C*). We next examined the effect of 2-h blocking of sCaLTs on the expansion of OL process arbors by live cell imaging using DIC microscopy. Here, OLs were treated with SFK96365 (10 μm) for 2 h and then imaged for 20 min using DIC. We found that OLs treated with SFK96365 showed essentially no extension of their processes during the 20-min live cell recording (Fig. 8*D*), whereas the control OL exhibited substantial extension of many of its processes (Fig. 8*D*, arrows). Further quantification shows that control OLs expanded 13.06 ± 6.7% (mean ± SD) of the total arbor area in a 20-min period, which was markedly reduced after 2-h exposure to SFK96365 (10 μm) or calcium-free buffer (Fig. 8*E*). Consistently, dynamic changes in F-actin structures in OL branches were also markedly reduced after 2-h exposure to calcium-free buffer as assessed by 20-min live cell fluorescence imaging of Ruby-Lifeact, a small probe for F-actin (Riedl et al., 2008; Fig. 8*F*). To determine whether sCaLTs are correlated with the actin-based protrusive activities, we co-transfected OLs with a genetically encoded calcium indicator Lck-GCaMP3, and Ruby-Lifeact, and performed time-lapse imaging on both two channels simultaneously. We observed a close association between sCaLTs and actin-based protrusive activity (Fig. 8*G*). Moreover, we found that sCaLTs often preceded actin-based protrusion. For example, in Figure 8*G*, we found an increased number of actin-based protrusion at location (i) was observed after the appearance of a sCaLT, but not at a nearby location (ii) where no sCaLT was seen (Fig. 8*G*). When we quantified this, we found that the formation of ∼70% (32 of 46) of protrusions correlated with sCaLTs from nearby branches. These data suggest that sCaLTs likely promote OL process outgrowth by promoting actin-based protrusive activities.

## Discussion

The ensheathment of axonal fibers by myelin membranes represents a crucial feature in the vertebrate brain that insulates axons for rapid propagation of nerve impulses, as well as to promote axonal survival (Baumann and Pham-Dinh, 2001; Nave, 2010). However, the molecular and cellular mechanisms underlying the transformation of bipolar OL precursor cells into mature myelinating OLs remain incompletely understood. Using an *in vitro* culture system, we have identified a novel form of spontaneous Ca^2+^ signals that are spatially restricted to the branches of OLs and are positively correlated with OL development. Furthermore, we have provided evidence that sCaLTs are primarily generated through SOCE and Ca^2+^ release from internal Ca^2+^ stores. Finally, blocking sCaLTs impairs the development and maturation of OLs in culture. These findings suggest that spontaneously generated Ca^2+^ signals play an important role in OL differentiation and maturation.

Several previous studies have shown the expression of VGCCs in OLs as well as the elevation of [Ca^2+^]_i_ in response to ligand stimulation (Takeda et al., 1995; Pitman and Young, 2016). Genetic knock-out of L-type VGCC CaV1.2 in OLs impairs OPC migration, process elaboration, and myelination (Cheli et al., 2016; Santiago González et al., 2017). Furthermore, changes in the resting [Ca^2+^]_i_ have been associated with the production of MBP in OLs (Friess et al., 2016). Further studies have shown that OLs can respond to neuronal activity or injury with elevation in [Ca^2+^]_i_, suggesting that Ca^2+^ signaling may function in neuron-OL interactions. Recently, two studies documented spatiotemporally occurring Ca^2+^ transients in OLs and demonstrated their importance in myelin sheath formation in zebrafish (Baraban et al., 2018; Krasnow et al., 2018). However, both studies mainly focused on the elongation and retraction of the myelin sheath during axon enwrapment and the Ca^2+^ transients in these studies are mostly induced by neuronal activity. In particular, approximately half of these Ca^2+^transients were evoked by action potentials (Krasnow et al., 2018). It should be noted that Ca^2+^ transients were also observed in premyelinating OLs with highly elaborated branches (Krasnow et al., 2018), although whether these OLs had made contact with axons or received neuronal input remain unknown. Our study here focused on the early stages of OL development without neuronal involvement. Specifically, we show that developing OLs exhibit sCaLTs that are important for the extension and elaboration of branched processes in developing OLs. It should also be noted that the spatiotemporal dynamics of sCaLTs observed in our study are different from those observed in myelinating OLs in these two studies. In particular, the lack of somatic Ca^2+^ transients in our study distinguishes our sCaLTs from the Ca^2+^ transients observed in previous studies (Krasnow et al., 2018). Our findings thus add additional evidence of an important role for Ca^2+^ in OL development. Importantly, our study has identified a novel form of Ca^2+^ signal (i.e., sCaLTs) that is spontaneously generated, spatiotemporally restricted in discrete regions of OL processes, and independent of neuronal input. Significantly, our study has identified SOCE as a major contributor to sCaLTs, thus shedding new light on Ca^2+^ signaling mechanisms underlying oligodendroglial development.

As a part of the intracellular Ca^2+^ homeostasis machinery, the function of SOCE is well established in non-excitable cells and considered as a Ca^2+^ entry mechanism for refilling intracellular Ca^2+^ stores. Increasing evidence indicates that SOCE participates and mediates a wide range of Ca^2+^ signaling cascades that regulate various cellular events. For example, SOCE has been shown to function in the generation of local Ca^2+^ transients in nerve growth cones to regulate their motility (Shim et al., 2013). The molecular components of SOCE include STIM, Orai, and Transient Receptor Potential (TRP) channels (Cahalan, 2009; Lewis, 2011), of which STIM resides on the ER membrane to function as the ER Ca^2+^ sensor, whereas Orai and TRP channels are on the plasma membrane. In response to Ca^2+^ depletion, STIM1 oligomerizes and translocates to ER and plasma membrane junctions, where it interacts with and activates SOC channels that include TRP Canonical 1 (TRPC1) and Orai1 proteins (Cahalan, 2009; Lewis, 2011) to enable Ca^2+^ influx. Previous studies have shown that STIM, Orai, and TRP channels are expressed in OLs but the function of SOCE in these cells is not well understood. Our study provides evidence that SOCE plays an important role in generating sCaLTs and suggests that SOCE may function in OL development. Both Ry receptors and inositol trisphosphate receptors are expressed in OL progenitors (Haak et al., 2001). Our data support the notion that SOCE and Ca^2+^ release from internal stores work in concert to generate sCaLTs underlying OL development. It should be noted that our work concerning SOCE and internal Ca^2+^ release used specific pharmacological reagents to interfere with these pathways. Since SKF96365 could also act on other targets in addition to STIM1, specific siRNAs should be used in future studies to knock down these SOCE components to examine the function of SOCE in sCaLTs generation and OL development.

It remains to be determined how sCaLTs may function to regulate the development and differentiation of OLs in culture. Morphologically, the occurrence of sCaLTs reaches its peak at the same time that OLs undergoes extensive process outgrowth and branching. Previous studies in neurons have shown that local elevation in [Ca^2+^]_i_ can elicit local actin-based filopodia protrusions (Lau et al., 1999; Nimchinsky et al., 2002; Kalil and Dent, 2014). It is possible that increased occurrence of sCaLTs may promote local actin activities for increased sprouting of filopodia and branch formation. Recent studies demonstrate that actin assembly is required for OL process extension during differentiation, while actin disassembly and reassembly is required for myelin wrapping (Nawaz et al., 2015; Zuchero et al., 2015). Previous studies have showed that the actin cytoskeleton is sensitive to changes in calcium, which affect contractility, actin-severing proteins, actin-crosslinking proteins, and calmodulin-regulated enzymes, and that calcium regulates the assembly and disassembly of actin structures (Yin et al., 1981; Furukawa et al., 2003). We speculate that the sCaLTs might be correlated with the local activity of actin cytoskeleton. Further studies could focus on if and how local actin assembly and disassembly may be regulated by sCaLTs during OL development. The reduction of sCaLTs in OLs bearing MBP-positive membranes suggests a diminishing role for spontaneous Ca^2+^ signals in the late stage of OL development, such as during myelination. Given that Ca^2+^ is known to play a critical role in myelination *vivo* (Soliven, 2001; Cheli et al., 2015; Baraban et al., 2018; Krasnow et al., 2018), neuronal input could elicit Ca^2+^ signals that regulate the myelination process. The absence of neurons and the cell culture nature of our system do represent the limitation of our study. Nonetheless, our study in culture has enabled us to identify a novel Ca^2+^ mechanism involving spontaneous Ca^2+^ signals from SOCE and Ca^2+^ stores that regulate OL development. Future studies are needed to investigate the role of spontaneous Ca^2+^ signals in OL development *in vivo*.

## Synthesis

Reviewing Editor: Quentin Pittman, University of Calgary Decisions are customarily a result of the Reviewing Editor and the peer reviewers coming together and discussing their recommendations until a consensus is reached. When revisions are invited, a fact-based synthesis statement explaining their decision and outlining what is needed to prepare a revision will be listed below. The following reviewer(s) agreed to reveal their identity: David Attwell.

The reviewers both agree that your manuscript is substantially improved. There remain a few minor points for you to address as follows:

As requested earlier, the authors need to state in the title of the paper that this work was done in culture without neurons - insert “cultured” before “oligodendrocytes”.

At the end of the methods (statistics) I am unsure what “parametric data” means. Do you mean data that a statistical test showed followed the normal distribution and so could be analysed by parametric statistical tests?

My one remaining concern is how they analyzed F-actin dynamics. From their brief description is seems to me that differences in cell protrusion will account their measured differences in F-actin dynamics. While differences in F-actin dynamics likely account for differences in cell protrusion, their measurements do not directly indicated F-actin dynamics. I suggest they try a different analysis or they explain what they did more clearly. For example, if they did the same analysis of simply GFP expressing OLs, they should not get the same result (F-actin specific result).

Lines 234-236 and 416-422. Contrary to what is stated, Krasnow et al. did characterise Ca transients and their spatiotemporal properties before contact with neurons (see their Fig 1A) and as stated in line 421 50% of the transients in sheaths were independent of neuronal activity, not “most” of them.

Line 305: perhaps point out the contrast with Cheli et al.?

Lines 427-428: contrast with the data in Fig 1A of Krasnow et al. who did see somatic Ca transients before neuronal contact.
